# Class II pentalogy of Cantrell

**DOI:** 10.1186/s13104-015-1293-7

**Published:** 2015-07-29

**Authors:** Helga Naburi, Evelyne Assenga, Sonal Patel, Augustine Massawe, Karim Manji

**Affiliations:** Department of Paediatrics and Child Health, Muhimbili University of Health and Allied Sciences, Dar es Salaam, Tanzania

**Keywords:** Pentalogy of Cantrell, Ectopia cordis, Thoraco-abdominal syndrome

## Abstract

**Background:**

Pentalogy of Cantrell is a rare syndrome, first described by Cantrell and co-workers in 1958. The syndrome is characterized by the presence of five major congenital defects involving the diaphragm, abdominal wall, the diaphragmatic pericardium, lower sternum and various congenital intra-cardiac abnormalities. The syndrome has never been reported in Tanzania, although may have been reported from other African countries. Survival rate of the complete form of pentalogy of Cantrell is as low as 20%, but recent studies have reported normal growth achieved by 6 years of age where corrective surgeries were done; showing that surgical repair early in life is essential for survival.

**Case presentation:**

The African baby residing in Tanzania was referred from a district hospital on the second day of life. She was noted to have a huge omphalocele and ectopia cordis covered by a thin membrane, with bowels visible through the membrane and the cardiac impulse visible just below the epigastrium. Despite the physical anomaly, she appeared to saturate well in room air and had stable vitals. Her chest X-ray revealed the absence of the lower segments of the sternum and echocardiography showed multiple intra-cardiac defects. Based on these findings, the diagnosis of pentalogy of Cantrell was reached. On her fifth day of life, the neonate was noted to have signs of cardiac failure characterized by easy fatigability and restlessness during feeding. Cardiac failure treatment was initiated and she was discharged on parents’ request on the second week of life. Due to inadequate facilities to undertake this complex corrective surgery, arrangements were being made to refer her abroad. In the meantime, her growth and development was satisfactory until the age of 9 months, when she ran out of the medications and succumbed to death. Her parents could no longer afford transport cost to attend the monthly clinic visits, where the infant was getting free medication refill.

**Conclusions:**

The case reported here highlights that in resource limited settings; poor outcome in infants with complex congenital anomalies is a function of multiple factors. However, we believe that surgery would have averted mortality in this 9-month-old female infant. We hope to be able to manage these cases better in future following the recent establishment of cardiac surgery facilities at Muhimbili National Hospital.

## Background

Pentalogy of Cantrell is a rare congenital anomaly, first described by Cantrell et al. in 1958 [1]. It occurs in 5.5 infants per 1,000,000 live births, globally. It is defined by the presence of five major defects, involving (1) the midline supraumbilical abdominal wall, (2) lower sternum, (3) anterior diaphragm, (4) diaphragmatic pericardium and (5) the heart (intracardiac defects) [1].

The cause of pentalogy of Cantrell is not clearly known and it is unclear whether it represents an extreme spectrum of midline disorders, since it shares some features with an X-linked dominant midline defect (thoracoabdominal syndrome) and has been reported to co-exist with other midline disorders [2–9]. In addition to classic anomalies described by Cantrell et al., few cases have been reported in co-existence with other syndromes such as Edwards’s and Goltz–Gorlin syndrome [10–12]. Similarly, other structural anomalies including craniofacial [2, 13] (e.g. cleft palate, supernumerary nares), central nervous system [8, 14–16] (e.g. hydrocephalus and neural tube defects), skeletal [12, 17–19] and abdominal abnormalities have been reported [20–23].

Surgical intervention to repair the defects is the treatment of choice and successful surgery has been reported despite post-operative complications. The prognosis depends on the severity of the cardiac defects [24–29]. We are reporting this case because pentalogy of Cantrell has never been reported in Tanzania and we are highlighting the challenges we faced while managing this case.

## Case report

An African infant aged 9 months, residing in Tanzania was first seen at the age of 2 days in our newborn unit of Muhimbili National hospital. This infant had been referred from Rufiji District Hospital (about 173 km apart) because of an abnormally positioned heart and an abdominal wall defect. The defect was noticed by the mother soon after delivery and was confirmed by the attending midwives.

The baby was born normally at term after an uneventful pregnancy. There was no history of consanguinity or exposure to obviously recognized teratogenic substances. The mother was on routine hematinic as per the antenatal care program in Tanzania. However, there was no antenatal sonography done at anytime during her pregnancy. The father was 40 years old and the mother was 26 years old when the baby was born. Parents are healthy, with no family history suggestive of birth defects, and they are both African peasants residing in rural Tanzania.

On initial examination, the infant was alert, with no pallor, cyanosis or jaundice and the anthropometric examination was normal for gestational age.

**Fig. 1:**
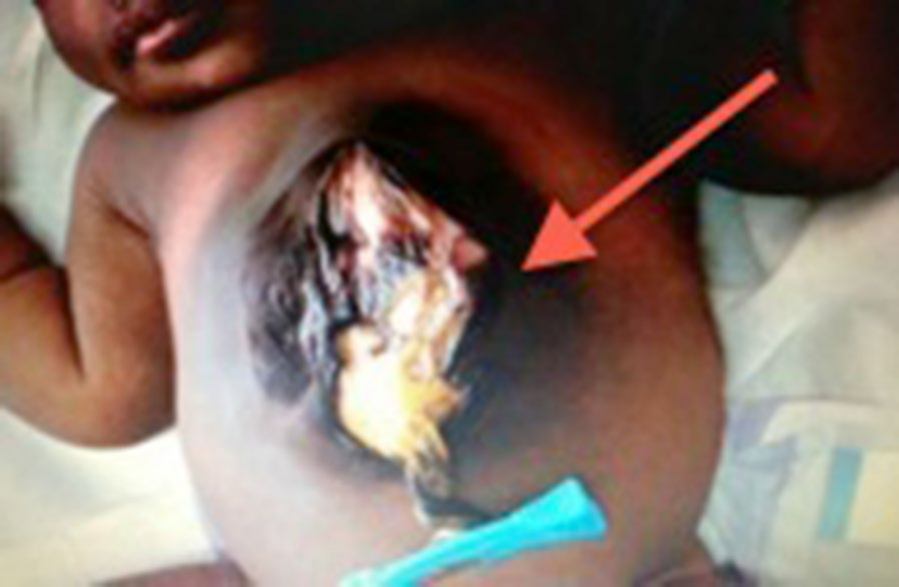
A large anterior abdominal wall defect, with a *pink colored* central area and *dark* surrounding skin.

There was an obvious lower chest wall defect with cardiac pulsation visible at the epigastrium covered by a thin membrane. The manubrium was palpable but the sternal body was not appreciated and there was a large omphalocele measuring 15 × 10 cm, extending proximally to the epigastrium, covered by a thin membrane and dark surrounding skin (Fig. 1). There were no other congenital malformations detected.

Her respiratory rate was 44 breaths per minute and she was saturating at 98% in room air.

She had a heart rate of 152 beats per minute, with the apex beat palpable over the epigastrium and a systolic thrill felt at the epigastrium. There was a diffuse pan systolic murmur grade 4/6 heard over the epigastrium and other systems were essentially normal.

**Fig. 2:**
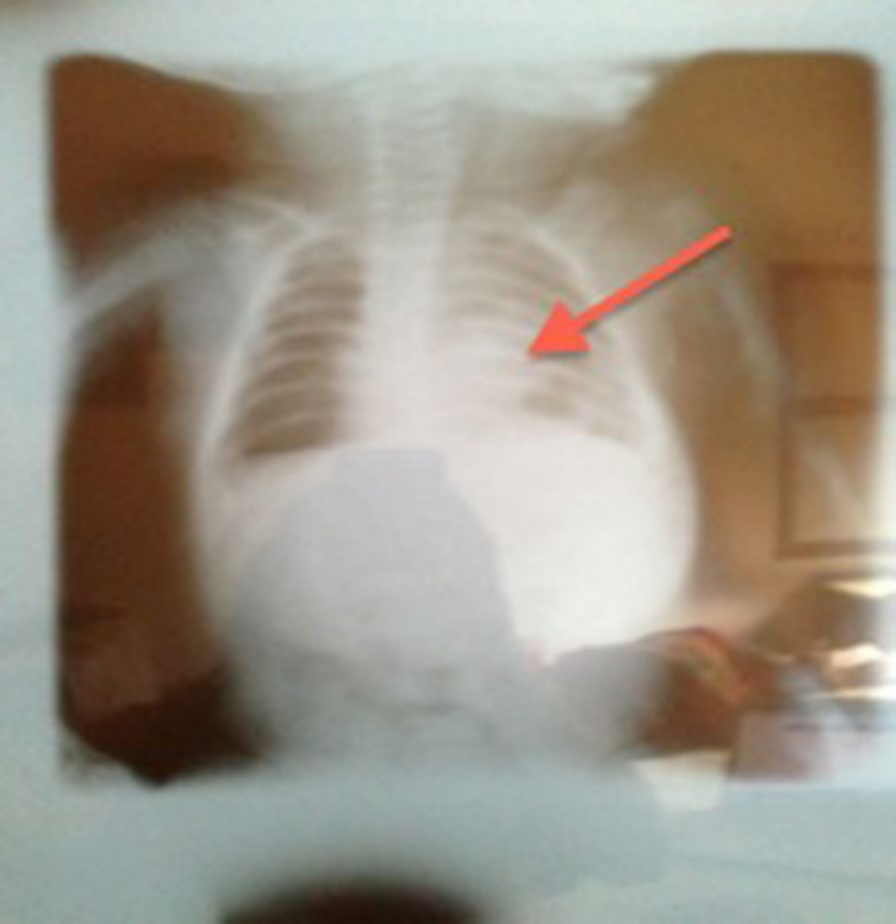
Chest X-ray of the patient taken within the first month of life, showing normal lung fields with distal displacement of the cardiac shadow.

Her chest radiograph showed a normal manubrium-sternum and lungs, with a displaced cardiac shadow (Fig. 2).

The electrocardiogram was not done because the machine was out of order. A transthoracic M-mode two-dimensional (2D) and Doppler echocardiogram were done using Toshiba Echo machine with a 5MHZ transducer. The report showed an intact pericardium, moderate to large ventricular septal defect (VSD) measuring 6 mm with a left to right shunt, a large atrial septal defect (ASD) measuring 7 mm, with a left to right shunt. Based on these findings a conclusion of complete atrioventricular canal defect was made.

Her abdomino-pelvic ultrasound showed an anterior abdominal wall herniation at the epigastrium with cardiac pulsation.

**Fig. 3:**
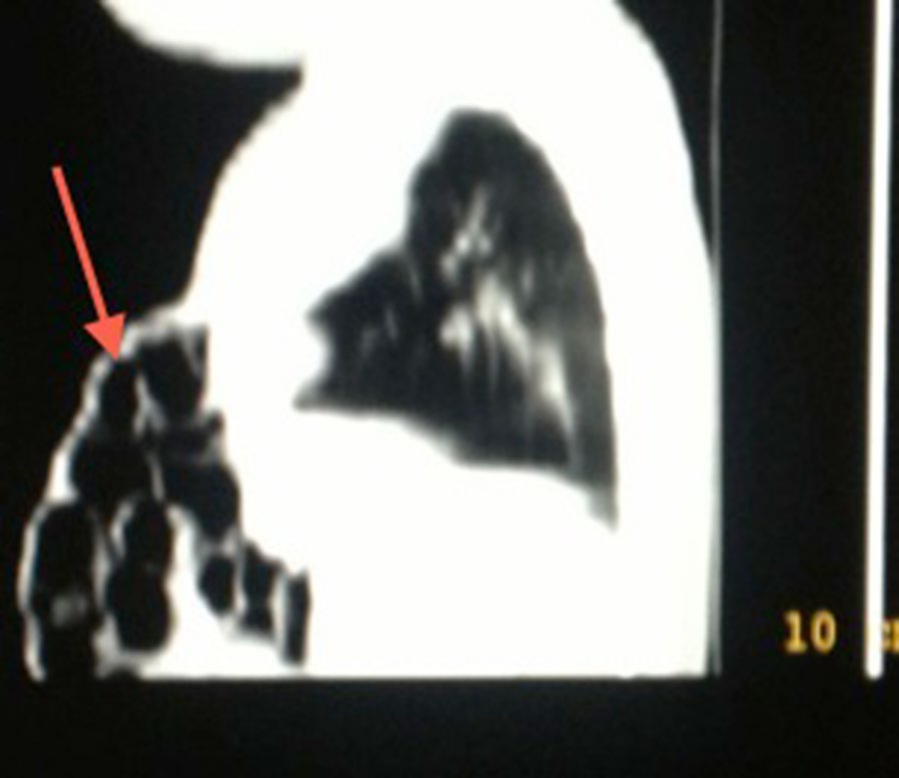
CT scan of the patient done during first month of life, showing loops of bowel protruding in the anterior abdominal wall defect (demonstrated by the *arrow*).

Computerized tomography (CT) scan findings showed the absence of the mid portion and distal segment of the sternum, presence of an anterior abdominal wall defect with protrusion of the bowel loops into the thoracic cavity and a cardiac shadow noted at the epigastrium (Fig. 3).

With the clinical presentation and investigation findings reported, we arrived at a diagnosis of class II pentalogy of Cantrell, based on the presence of four (midline supra-umbilical abdominal wall defect, a defect of the lower sternum, a deficiency of the anterior diaphragm and congenital intra-cardiac defects) out of five features of the Tayoma classification [30].

**Fig. 4:**
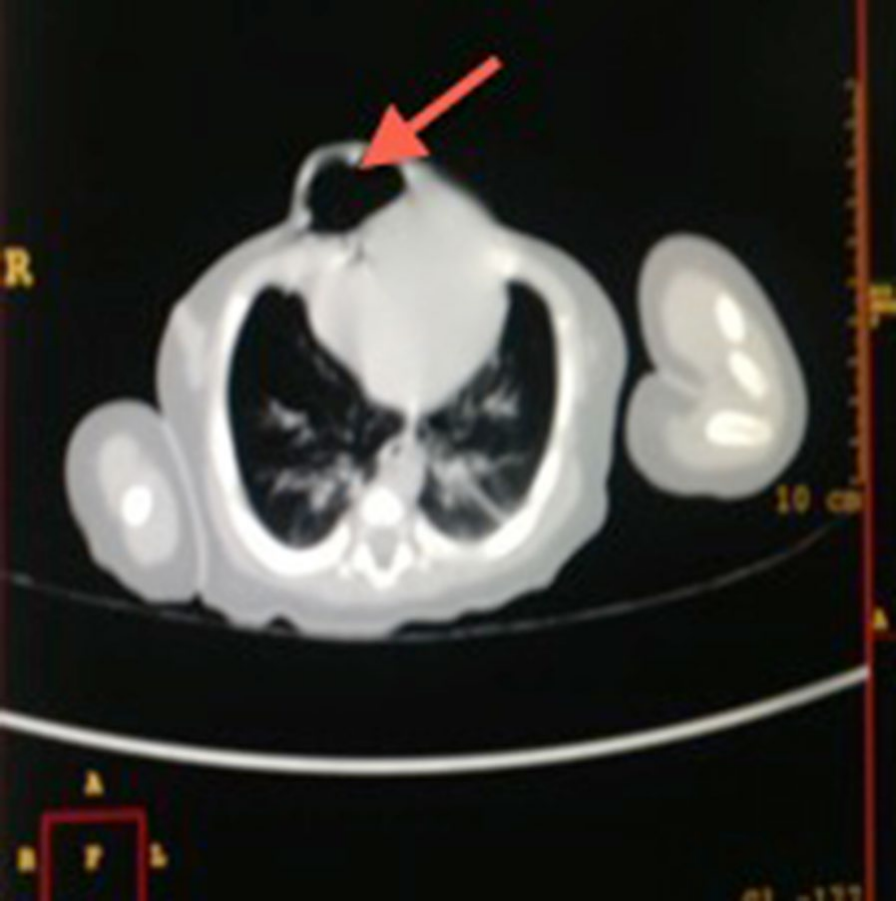
Axial CT scan of the patient showing the heart bulging though the anterior abdominal wall defect proximal to air filled bowel loops.

The infant was treated with furosemide 1 mg/kg twice a day and spironolactone 1 mg/kg twice a day to control the heart failure, while awaiting corrective surgical treatment. Parents requested for discharge on financial grounds, as both were peasants and had no other source of income. We continued to follow up the infant monthly in our outpatient clinic, while processing the referral abroad for corrective surgery. During these visits the medication refill was offered free of charge and from parents’ self-report it was determined that adherence to medication was good. Her milestones were normal and growth adequate until the age of 8 months when she missed a clinic visit and ran out of medication for about 1 week. Parents could not afford the transport cost to bring the infant for the follow up clinic visits; hence she could not get a refill. While out of medication, the distress during breastfeeding returned, and parents had to borrow money to bring the child to the hospital. As the infant resumed her medication, the symptoms improved tremendously. However, at the age of 9 months before plans for referral abroad for corrective cardiac surgery could be finalized, she died suddenly at home. The immediate cause of death could not be determined since an autopsy was not done and the information was received from parents a week after she passed away. The limited facilities for complex paediatric cardiac surgery in our hospital and parents’ financial constraints were among the factors that contributed to the early death of the infant (Fig. 4).

## Discussion

Pentalogy of Cantrell was first described by Cantrell, et al. in 1958, based on the presence of a midline supra-umbilical abdominal wall defect, a defect of the lower sternum, a deficiency of the anterior diaphragm, a defect in the diaphragmatic pericardium and congenital intra-cardiac defects [1].

Cantrell et al. suggested that the pattern of malformation in this syndrome might be a result of defective formation and differentiation of the ventral mesoderm at about 14–18 days of gestation [1]. Failure of the development of a mesoderm segment results in diaphragmatic, pericardial and intracardiac defects; whereas sternal and abdominal wall defects result from failure of paired primordial structures to migrate to their appropriate locations [1].

Based on the 60 cases reviewed by Tayoma, the syndrome is classified as complete or incomplete according to the number of malformations present [30].

*Class 1* definite diagnosis and all five defects are present.

*Class 2* probable diagnosis, if the four defects are present including: intracardiac and ventral abdominal wall defects.

*Class 3* incomplete expression, with the various combinations of defects present and includes a sternal abnormality.

A recent review of cases of pentalogy of Cantrell reported from 1998 to 2007 shows the complete form to be more common, accounting for more than half of the cases. Of the 58 patients reviewed by Van Horn et al., 39 patients had intracardiac anomalies [31]. Congenital intracardiac anomalies are consistent elements of this syndrome, and Cantrell et al. reported VSD in almost all cases, ASD in about half of cases, tetralogy of Fallot and left ventricular diverticulum each present in one-fifth of the patients [1].

Based on our investigation results we classified the patient to have the class II form of the syndrome due to the presence of four defects including: midline supraumbilical abdominal wall defect, a defect of the lower sternum and complex intracardiac defects, with an intact pericardium. Since it is very rare to have an intact pericardium and ectopia cordis we may have missed the pericardial defect, which would put the patient in the class I form of the syndrome. However, Pachajoa et al. had also described a case with an absent diaphragmatic pericardial defect in presence of a midline defect, upper abdominal wall abnormality, lower sternal defect and an anterior diaphragmatic defect [32]. The intracardiac defects detected in this patient included VSD and ASD. The presence of these defects is similar to the case described by Ootaki et al., however, in their case the patient had the complete form of Pentalogy of Cantrell and successful surgery was done at age of 7 months [25]. Similarly Wen et al. described an incomplete form of pentalogy of Cantrell with ASD and VSD. However, contrary to the case presented here, double outlet right ventricle, transposition of great arteries and pulmonary stenosis were additional findings and it was not reported if surgery was done; but the patient was last seen at the age of 4.7 years [33].

Our case could have had a relatively better outcome since she had an incomplete form of the syndrome. Previous reports suggest that, mortality is higher in infants with the complete form and associated extra cardiac anomalies whilst patients with ectopia cordis with intracardiac defects have a favorable outcome [31, 34].

With advancement in medical knowledge and technology, patients with complex intracardiac defects can survive longer beyond 6 years of age with normal growth and development [27, 33–35]. A child with the incomplete form of pentalogy of Cantrell without intracardiac defects was reported to have corrective surgery at 11 years of age and a more mild form of the syndrome was seen in an adult case [36, 37].

In the past 5 years, 23 new cases of pentalogy of Cantrell were published in different English journals and could be retrieved through the PubMed database. Out of these cases approximately 21 (91%) were reported to have survived by the time their case reports were written. In these cases the median age at their last contact was 10 months (ranging between 4 days to 5 years) [12, 25, 27, 33–47].

Prenatal diagnosis can be done using qualified and experienced obstetric ultra sonographers as early as 10 weeks of gestation using the traditional two dimensional (2D) imaging sonography; at which stage an omphalocele and ectopia cordis are common findings [48–54]. While a 3D imaging sonography may be required for the diagnosis of certain fetal anomalies, the diagnosis of pentalogy of Cantrell can sufficiently be made with a traditional 2D imaging sonography [49, 51, 52, 54].

Obstetric ultrasound is indicated as a standard of care for routine evaluation of pregnant women in Tanzania, where majority of facilities have 2D imaging sonography machines. However, in our case prenatal ultrasound was not done at any point during the pregnancy, thus we missed the opportunity for prenatal counseling and early referral of this child.

## Conclusions

The case reported here highlights that poor outcome in infants with pentalogy of Cantrell is inevitable if early intervention with corrective cardiac surgery for these patients is not offered. Palliation with cardiac failure treatment requires good adherence, which may be may be affected by various factors such as socioeconomics. We believe that early surgery would have averted mortality in this infant and this has stimulated us to take a more aggressive approach with a 2-month old infant with a similar condition and for who we were able to fast track the referral arrangements. We hope to be able to manage these cases better in future following the recent establishment of the cardiac surgery facilities in our hospital.

## Abbreviations

2D: two dimensional
3D: three-dimensional
ASD: atrial septal defect
CT: computerized tomography
VSD: ventricular septal defect
MHZ: megahertz
